# Supplementary material for: Wing structure and neural encoding jointly determine sensing strategies in insect flight
Source: PLoS Comput Biol. 2021 Aug 11;17(8):e1009195. doi: 10.1371/journal.pcbi.1009195 (PMC8382179; doi:10.1371/journal.pcbi.1009195)
Supplement: S1 Appendix — (PDF) [file pcbi.1009195.s001.pdf]

S1 Appendix

Effects of changing the frequency of the linear filter

The filter frequency in this work is chosen to correspond to previous work [23], which in turn reflects experimentally measured linear filters [14]. We assess the effects of changing the filter frequency while holding other parameters of neural encoding constant. Nonlinear threshold is held at 0.1, and all other parameters are as indicated in the Methods. Filters of different frequencies are normalized by the sum of squared values at all time points.

Interestingly, classification accuracy depends most strongly on stiffness for this value reflecting experimental observations (indicated by gray box in Fig A, filter frequency parameter = 1, corresponding to $\omega = {}^{1}\!/_{2\pi}\ \mathrm{ms}^{-1}$) compared to higher and lower frequency filters. For higher frequency filters, performance generally does not rise above chance, due to the fact that strain signals contain little to no power at these frequencies. At lower frequencies and when the filter is entirely flat (performing an average over the length of the filter) classification accuracy is nearly 100% across the range of wing stiffness values tested. The experimentally observed frequency content of linear filters in mechanosensory neurons therefore appears to interact particularly strongly with other features of the system (stiffness and neural threshold).

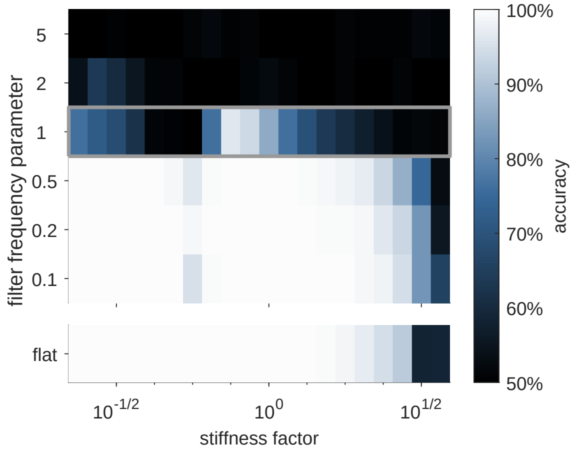

Fig A. Effects of changing linear filter frequency. Accuracy in detecting rotation in the yaw axis as a function of filter frequency and wing stiffness. Gray box indicates filter frequency used in the rest of the paper. Neural threshold is held constant at 0.1.

Sensing with two wings

Because the wings we study are symmetric in both shape and stiffness, sensing strategies for two wings are expected to be approximately the same. However, we wished to determine whether accuracy would be significantly impacted by using two wings to perform the yaw sensing task. We hold the total number of sensors constant at 10, though now sensors may be distributed across the two wings. If there are a small number of locations that are particularly informative, addition of the second wing could significantly boost performance. However, we see only modest gains in accuracy for a subset of stiffness/neural threshold combinations. For simplicity we therefore focus on sensing in a single wing.

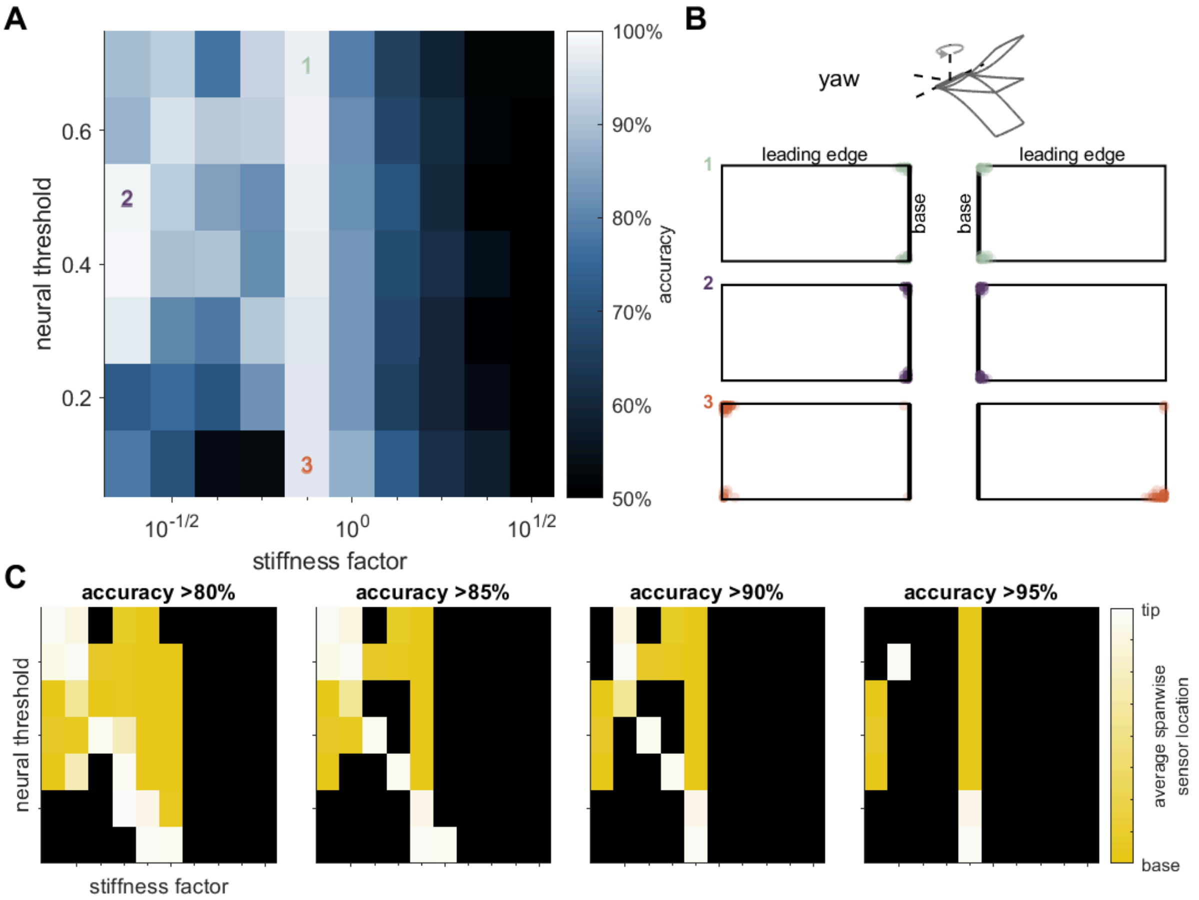

Fig B. Yaw detection performance and sensor locations are similar with two wings compared to one. A: Accuracy as a function of neural threshold and wing stiffness. B: Optimal sensor locations of 10 best sensors across both wings, overlaid for 10 different simulated data sets. Neural threshold and wing stiffness of each panel are indicated by the corresponding colored dot in A. C: Optimal sensor locations in the spanwise direction from wing base (yellow) to wing tip (white) as a function of neural threshold and wing stiffness. Each panel shows results for a different accuracy cutoff. Black indicates parameter combinations that fall below the cutoff.

Spiking vs. non-spiking sensors

Previous work considered optimal sensing strategies for non-spiking neurons [23]. For comparison, we assessed classification accuracy in non-spiking neurons over the range of stiffness values and neural thresholds used in the rest of the present study. For non-spiking sensors, responses were simulated at a temporal resolution of 1 ms, and classification is based on instantaneous probability of firing at each time point. Two interesting differences emerge between non-spiking and spiking sensors: (1) for yaw detection, non-spiking sensors perform best for stiff wings, while spiking sensors perform best for compliant wings; and (2) accuracy does not rise above chance for either pitch or roll detection in non-spiking neurons.

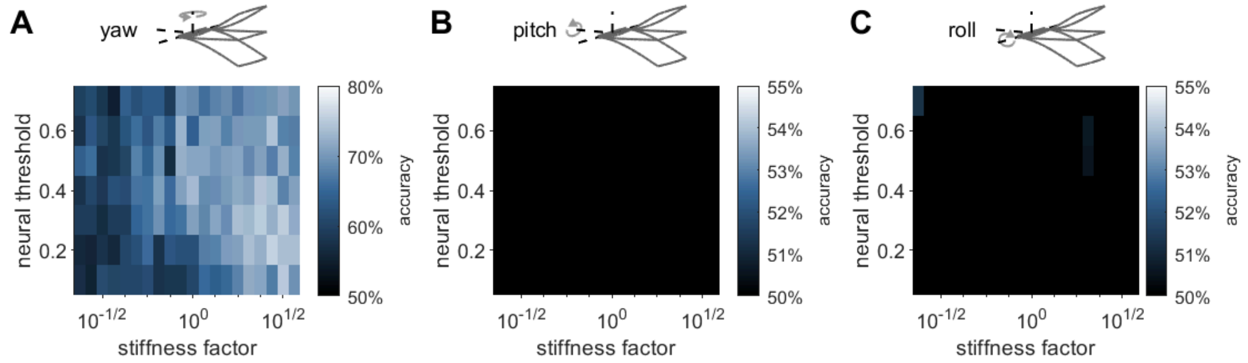

Fig C. Classification accuracy for non-spiking sensors. A–C: Classification accuracy for yaw, pitch, and roll detection, respectively.

Simultaneous classification of yaw, pitch, and roll rotation

Rather than showing fundamentally different trends, classification accuracy and sensor locations generally reflect a mixture of the three individual classification tasks (Fig D). Accuracy fails to rise above 65% for any combination of wing stiffness and neural threshold (Panel A in Fig D), reflecting a failure to accurately classify all four conditions. As expected from the individual axis classification tasks, pitch is most commonly confounded with the flapping only condition (Panel D in Fig D). Sensor locations show highest concentrations — as observed for yaw — and dispersed locations — as observed for roll and pitch — near the wing base (Panel B in Fig D), although sensor placement is more varied than in any of the individual classification tasks. Interestingly, performance in the four-way classification task is reasonably well-predicted by the average of all two-way classifications (subject to an overall offset and scaling; Fig E).

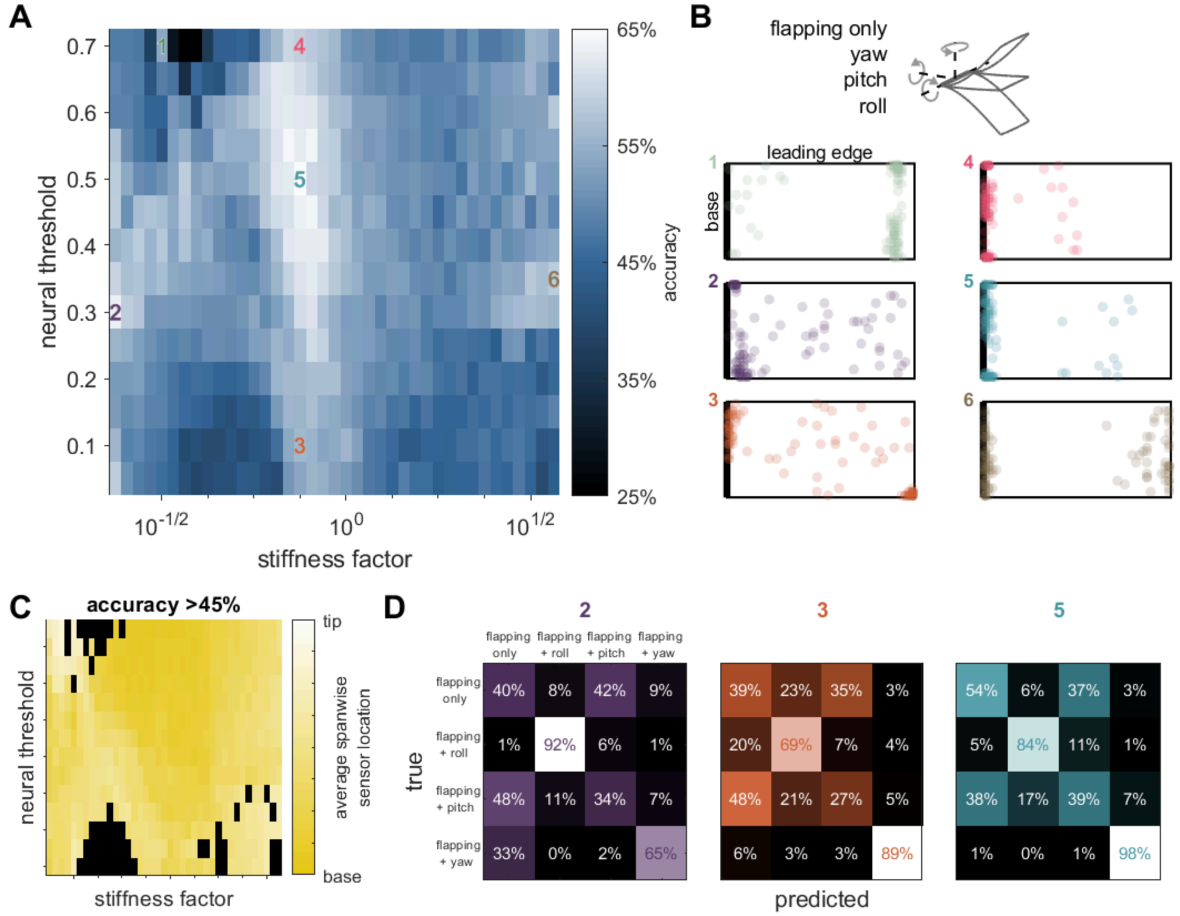

Fig D. Four-way classification of flapping only, rotation in yaw, and rotation in pitch. A: Accuracy as a function of neural threshold and wing stiffness. Note that chance performance is 25%. B: Optimal sensor locations of 10 best sensors, overlaid for 10 different data sets. Neural threshold and wing stiffness of each panel are indicated by the corresponding colored number in A. Top schematic indicates the possible axes of rotation. C: Optimal sensor locations in the spanwise direction from wing base (yellow) to wing tip (white) as a function of neural threshold and wing stiffness. Each panel shows results for a different accuracy cutoff. Black indicates parameter combinations that fall below the cutoff. D: Confusion matrices indicating detailed classification errors for each of three corresponding numbered points in A. Rows are normalized to add to 100%.

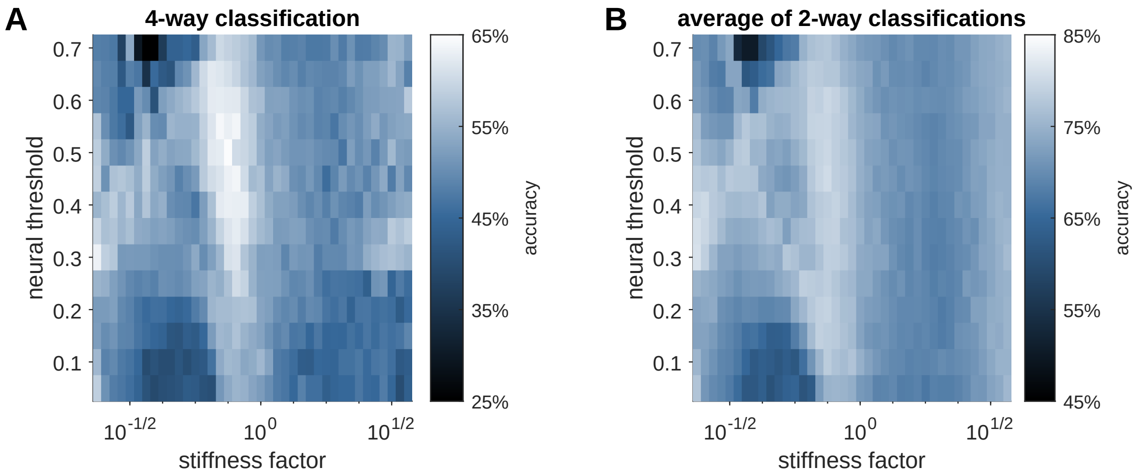

Fig E. Accuracy on four-way classification is well predicted by the average accuracy across all two-way classification tasks. A: Classification accuracy for four-way classification of flapping only, yaw rotation, pitch rotation, and roll rotation. B: Average classification accuracy across all individual two-way tasks: (1) flapping only vs. flapping + yaw; (2) flapping only vs. flapping + pitch; and (3) flapping only vs. flapping + roll.

Sensing strategies for rotation about an intermediate axis

To determine whether sensing strategies would be fundamentally different for rotation about an intermediate axis, we performed a separate set of optimizations to identify rotation about an axis intermediate between yaw and roll (equivalent to simultaneous rotation about the yaw and roll axes). The magnitude of rotation about this intermediate axis was identical to elsewhere in the present work (10 rad/s). Rather than showing fundamental differences in performance or sensing strategies, results reflect a combination of the strategies for sensing yaw only and pitch only. Accuracy is high across a larger range of the stiffness factor/neural threshold space than for either yaw or pitch alone, with stiffer wings showing sensing strategies (all base sensors) and accuracy similar to roll detection and more compliant wings showing sensing strategies (either tip or base sensors) and accuracy similar to yaw detection (Fig F). For some parameter combinations, sensing strategies reflect a combination of the individual yaw and roll strategies (e.g., #2 in Fig F shows both base and tip sensors).

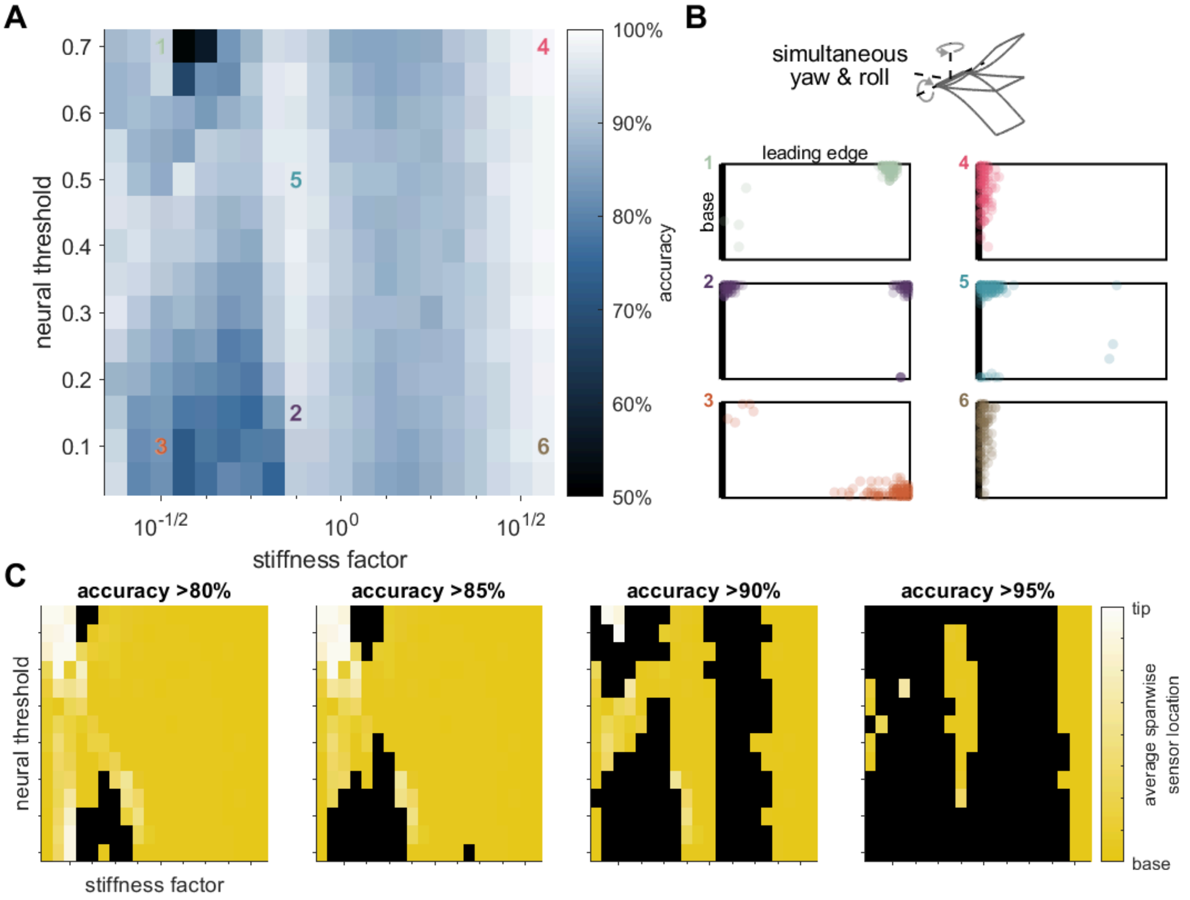

Fig F. Sensing simultaneous yaw and roll rotation. A: Accuracy as a function of neural threshold and wing stiffness. B: Optimal sensor locations of 10 best sensors, overlaid for 10 different data sets. Neural threshold and wing stiffness of each panel are indicated by the corresponding colored number in A. C: Optimal sensor locations in the spanwise direction from wing base (yellow) to wing tip (white) as a function of neural threshold and wing stiffness. Each panel shows results for a different accuracy cutoff. Black indicates parameter combinations that fall below the cutoff.

Constrained sensor locations to evaluate base vs. tip encoding strategies

We wished to determine how much performance would suffer if sensors were restricted to either the proximal or distal half of the wing for the yaw detection task. This has the effect of forcing sensors to fall at the wing tip when base sensors are optimal and vice versa. Although optimal sensor locations change abruptly from base to tip, accuracy for a given sensing strategy (i.e., base or tip) generally falls off more gradually in these regions of transition.

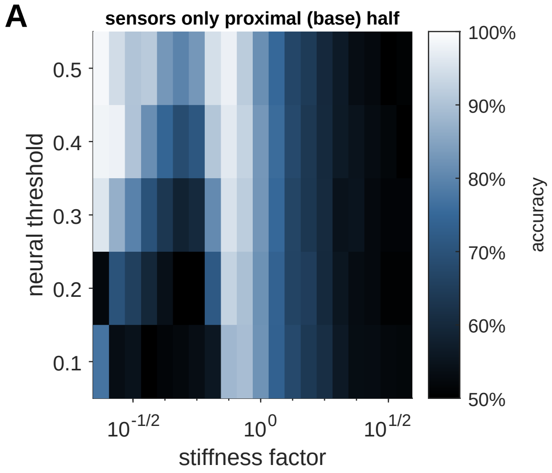

Fig G. Classification accuracy for yaw rotation with sensors location is constrained. A: Classification accuracy when sensors are restricted to the proximal half of the wing. B: Classification accuracy when sensors are restricted to the distal half of the wing.

Classifying rotation with chordwise strain

Performance is far lower if classification is based on chordwise strain rather than spanwise strain. For classifying rotation in the yaw axis (0 vs 10 rad/s), performance does not rise above chance. Results for classifying rotation in the pitch axis show a small drop in performance and, interestingly, a shift of optimal sensor locations from base to tip compared to classification with spanwise strain (Fig H; compare to Fig 4C and 4D).

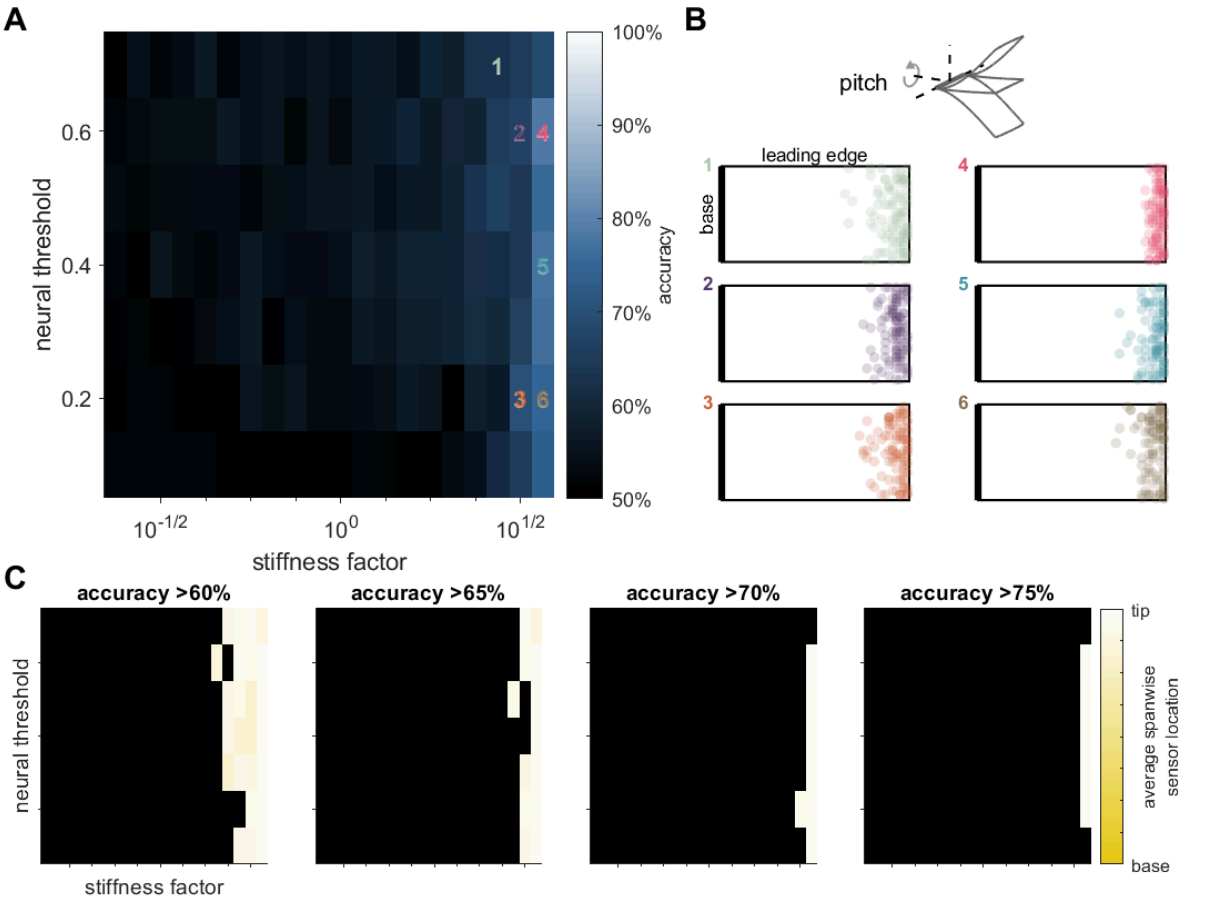

Fig H. Detecting rotation with chordwise strain reduces accuracy and changes locations of optimal sensors. A: Accuracy as a function of neural threshold and wing stiffness. B: Optimal sensor locations of 10 best sensors, overlaid for 20 different data sets. Neural threshold and wing stiffness of each panel are indicated by the corresponding colored number in A. *Top:* Schematic indicates the axis of rotation. C: Optimal sensor locations in the spanwise direction from wing base (yellow) to wing tip (white) as a function of neural threshold and wing stiffness. Each panel shows results for a different accuracy cutoff. Black indicates parameter combinations that fall below the cutoff. Note the overall lower accuracy and change in optimal sensor locations compared to Fig 4C and 4D, where spanwise strain is used for classification.

Performance evaluation with a nonlinear classifier

To determine the effects of our choice of linear classifier on our results, we also test the effects of using a nonlinear classifier to determine accuracy. As before, LDA is used to find the best projection vector w_c for the non-standardized test data for only the top 10 sensors. Boundaries are then drawn based on the Gaussian approximations of each category projected onto w_c. Generally only one boundary is used, corresponding to the intersection of the two Gaussian approximations, though in cases where one category has zero variance or variance is both categories is large, resulting in two intersections of the Gaussian distributions, two boundaries are used. We find that this results in only negligible changes to the overall accuracy (Fig I). Note that sensor locations remain identical to those in Fig 3, as only the classification scheme has changed, while the method of finding optimal sensor locations remains the same as before.

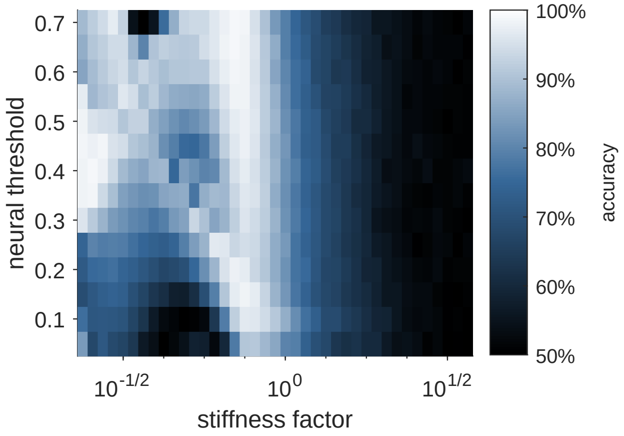

Fig I. Accuracy of detection of rotation in the yaw axis with a nonlinear classifier. Task accuracy is indistinguishable from a linear classifier.

Effects of changing the phase of the wingstroke that is defined as time 0

Because classification is based on the time to first spike within each wingstroke, it is possible that the phase of the wingstroke that is defined as t=0 may affect our results. In the above work, time zero is roughly the time of the greatest increase in wing strain over the course of the wingstroke. To test the impact of this, we shift the time defined as time zero by one half wingstroke (20 ms).

The results of this change have small effects on overall accuracy and optimal sensor locations (Fig J). Though there are slight differences in sensor locations for different neural threshold/stiffness factor combinations, the same distinct regions remain with sensors concentrated at either the wing base corners or at corners or edges near the wing tip.

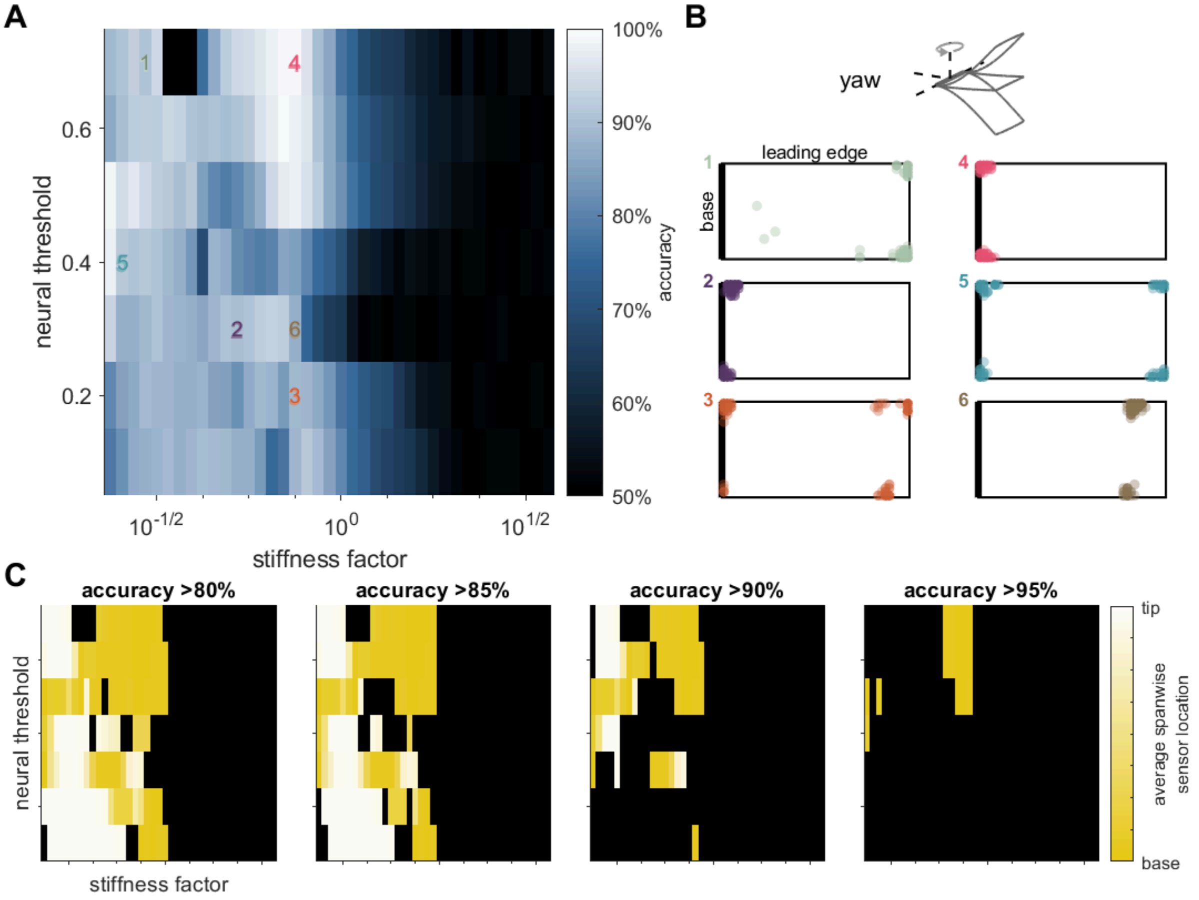

Fig J. Effects of changing the defined beginning of the wingstroke by 20 ms. A: Accuracy as a function of neural threshold and wing stiffness. B: Optimal sensor locations of 10 best sensors, overlaid for 20 different data sets. Neural threshold and wing stiffness of each panel are indicated by the corresponding colored number in A. Top schematic indicates the axis of rotation. C: Optimal sensor locations in the spanwise direction from wing base (yellow) to wing tip (white) as a function of neural threshold and wing stiffness. Each panel shows results for a different accuracy cutoff. Black indicates parameter combinations that fall below the cutoff.
