# Supplementary material for: Wing structure and neural encoding jointly determine sensing strategies in insect flight
Source: PLoS Comput Biol. 2021 Aug 11;17(8):e1009195. doi: 10.1371/journal.pcbi.1009195 (PMC8382179; doi:10.1371/journal.pcbi.1009195)
Supplement: Attachment — Submitted filename: AIW_PLOSCB_ResponseToReviewers.pdf [file pcbi.1009195.s002.pdf]

Responses to Reviewers

We thank the three reviewers for their supportive comments, thoughtful questions, and incisive suggestions, which have prompted us to make substantial changes to the manuscript.

In addition to clarifying several points in the paper, we have also performed a number of additional analyses in direct response to the reviewers' comments, which we feel have added important additional insights to the manuscript. The key revisions we undertook include:

1. A set of new analyses of optimal sensor locations for rotation about the roll axis, thus completing the paper's results for sensor locations in each of the 3 canonical axes of rotation (yaw, pitch, and roll).
2. Rewording of the Introduction and substantial restructuring of the Discussion to clarify the interpretations and connections of our computational modeling study, with all of its simplifying assumptions, to biological insect flight.

Below are our point-by-point responses to each reviewer's comments. In addition, we submit an annotated version of the changes we have made in this revised manuscript, where edits and additions to the paper are highlighted. All line numbers below correspond to the clean copy of the revised manuscript.

Reviewer #1:

The manuscript by Weber et al. used a numerical approach to study the effect of wing stiffness, neural encoding and strain-sensor position on the ability to distinguish between body rotation and flapping using the strain sensors data. The wing is flapped and parameterized as a hawkmoth wing and the analysis of the strain data is bio-(neural)-inspired. The manuscript is well written. The modelling approach is valid and based on empirical data, the work described is novel and the topic – effect of wing mechanical properties on sensing – is fascinating. For these reasons I don't have a problem with publication, but I do have a fundamental issue with the basic approach.

As the authors rightfully mention, this work is a first step towards understanding how wing structure determine rotation sensory (line 183). As a first step, I see the value of performing the analysis on a very simple shape (a flapped plate, lines 287-292) to isolate parameters (wing stiffness), but I resent the attempt to portray this shape as a "computational model of an insect wing" (line 185). The thing is, that in addition to having veins and membrane the insect wing has non uniform thickness, a different planform (particularly relevant for the sensors at the wing base) and non-uniform stiffness. The SI section re-analyzed the classification with chord-wise strain and gave quite different results (mainly for Yaw) but most of the insect wing-veins are neither parallel to the span or the chord. While some of these limitations are acknowledged in the Discussion, the question remains concerning to the relevance of the simulation results (sensor position, minimal number of sensors, accuracy) to insect flight. The

results are more of a 'proof of concept' of an idea rather than describing sensing in insects.

We thank the reviewer for pointing out this very important caveat of our approach, with which we fully agree. In the updated manuscript, we have made extensive revisions to the text to clarify what our approach does and does not do, especially with respect to the many assumptions and simplifications we have made in order to make the models and analyses computationally tractable.

As the reviewer rightly points out, our model is highly abstracted from insect wings and lacks many of the features (e.g., veins, shape, sensor orientation) that likely play an important role in sensing in biological systems. For this reason, in our initial manuscript, we refrained from making direct comparisons between our results and sensor placement in insects. We appreciate the concern about describing this as a "model of an insect wing" and have revised multiple descriptions in the manuscript so as not to overstate this comparison, e.g.:

- Abstract: "a computational wing model with varying flexural stiffness~~ inspired by the hawkmoth *Manduca sexta*~~"
- end of Introduction: "We use a simple model based on *Manduca sexta*..." → "We use a simple model, with wing size and stiffness based on the wings of *Manduca sexta*…"
- beginning of Discussion: "in a computational model of an insect wing" → "in a computational model of a flapping wing"

We have also expanded our explanation of the limitations of our approach and clarified what we think this model can tell us about useful strategies for mechanosensor placement in insects (Discussion, lines 240-301):

> **Strain sensor placement in insect wings**
>
> Insect wings exhibit widely varying patterns of mechanosensor placement on their wings [47-51]. Even so, we have little understanding of the functional consequences of this diversity. Moreover, comparison between species is complicated by the vast differences in wing morphology and stiffness [29, 52]. Despite these differences, some generalizations can be made about the placement of strain sensors (campaniform sensilla). Perhaps the most striking commonality across diverse taxa is the relative abundance of campaniform sensilla near the wing base [47-49, 51, 53-55]. Taken together, the results in the present work suggest that clustering sensors at the wing base may serve as a catch-all strategy, favorable across a wide range of wing and neural encoding properties to detect rotations about multiple axes (Figs 3,4; Supp Fig 6).
>
> **Additional features driving optimal sensor placement**
>
> Insect species vary widely in features that are likely to play a role in determining effective mechanosensor placement on the wings: wing size, shape, and venation pattern, among others. Understanding the functional consequences of these different

features and how they impact spatiotemporal patterns of strain on the wing will be necessary for elucidating principles guiding the differing placement of sensory structures across diverse insect taxa. Answering these questions will require different computational models that allow more flexible manipulation of these variables. For example, models based on the finite element method (FEM) support more realistic wing geometry, wing venation patterns, and non-uniform stiffness [29, 49]. The consequences of many of these properties have been previously explored in a variety of models, although none from a sensing perspective [18, 19, 29, 56, 57]. More biologically realistic features will result in more complex spatiotemporal patterns of wing strain, likely driving sensor placement to be far more complex and varied than those uncovered in the current study.

The wingstroke trajectory and kinematics may play a particularly important role in determining optimal sensor placement. In the current work, we see that the wingstroke, modeled as a sum of sines as in previous work [23, 31], drives two distinct response events over the course of single wingbeat (Fig 2). Interestingly, these response events interact with spike timing precision in sometimes counterintuitive ways, such that lower spike timing precision can sometimes yield greater discriminability. More complex wingstroke kinematics likely play an important role in determining the phase (or phases) within a wingstroke when each neuron is most sensitive. The trajectory of the wingstroke may also be used as a mechanism for active sensing, in which the animal modifies its motor output to acquire more useful sensory information [58-62]. Understanding the consequences of varying motor output for incoming sensory information will be crucial to understanding sensing strategies in flight.

Although we varied neural encoding properties (nonlinear threshold and filter frequency, Fig 3, Supp Fig 1), all sensors on a given wing have identical encoding properties. Previous work suggests that there are likely two sub-populations of strain-sensitive neurons on the wings of moths [63]. It is possible that a wing with different sensor types may need fewer sensors and adopt a qualitatively different encoding strategy. Additionally, selectivity to multiple features has been demonstrated in neurons in multiple sensory systems, including strain-sensitive neurons in the halteres of crane flies [6, 64]. Allowing individual sensors to respond to multiple stimulus features may also allow for fewer sensors and different sensor positioning. However, it has recently been suggested that all strain-sensitive neurons on insect wings may, in fact, respond similarly in the context of flight (i.e., for rapidly fluctuating stimuli), suggesting that sensor placement may be the primary determinant of information encoded by a given sensor [65].

The current work investigates sensing strategies by finding solutions to a set of optimization tasks. However, biological systems need not operate at any true optimum, but may instead simply need to be “good enough" or robust to failure [66]. Solving for optimal sensor locations likely explains some of the features of the present study, for example, the abrupt shift between wing base and wing tip sensing strategies for yaw detection (Fig 3). If sensor locations are restricted to either the distal or the proximal

half of the wing, sensor performance does not fall off sharply at these same transitions (Supp Fig 7). We investigated robustness of the optimal sensing strategies in the current study and found little difference between different sensor properties (locations or threshold), suggesting that a need for robustness would not favor one of these strategies over another. Exploring the range of sensing strategies that meet some (sub-optimal) performance criterion may be an interesting area of future work.

Regarding the specific point about mechanosensor orientation: we are keenly interested in this as well! Given the simplifications in the current model, particularly its symmetry and uniform stiffness, it generally lacks the features that would allow us to tackle this set of questions. We have therefore chosen to focus on spanwise strain sensing in this model, as the one-dimensional flapping motion produces the largest strain in the spanwise direction and, from a functional perspective, this is the most relevant direction for reading out information about body rotations. In actual insect wings, with large asymmetries, complex 3D geometries, and stiffness gradients along both the spanwise and chordwise directions, strain profiles will be vastly more complex than we have simulated, and intermediate sensor orientations (and differing orientations over the wing surface) are likely beneficial for extracting relevant strain information. We are excited to pursue this as a direction for future work that makes use of different modeling strategies (i.e., finite element methods, in preparation).

Minor comments

Is there a particular reason why roll is not considered or even mentioned?

Thank you for bringing up this new direction — we've decided to roll with it. In this revision, we have now included a set of new analyses on roll detection and arrived at some new results.

Based on some preliminary work in non-spiking sensors (similar to Supp Fig 3C), we had previously believed that detecting roll would be a much more challenging task than detecting pitch or yaw because the deformation modes are more similar to, and weaker than, those produced by the motion of wing flapping. Prompted by your question, as well as the suggestion of Reviewer #2, we have revisited roll detection in a more systematic way and have found that roll can indeed be detected with high accuracy. We have added these new results in the revised manuscript (Figs 4-5; Supp Figs 3-6) and believe that this strengthens the work by providing a more complete picture of rotation detection. Revised Figs 4-5 are copied here:

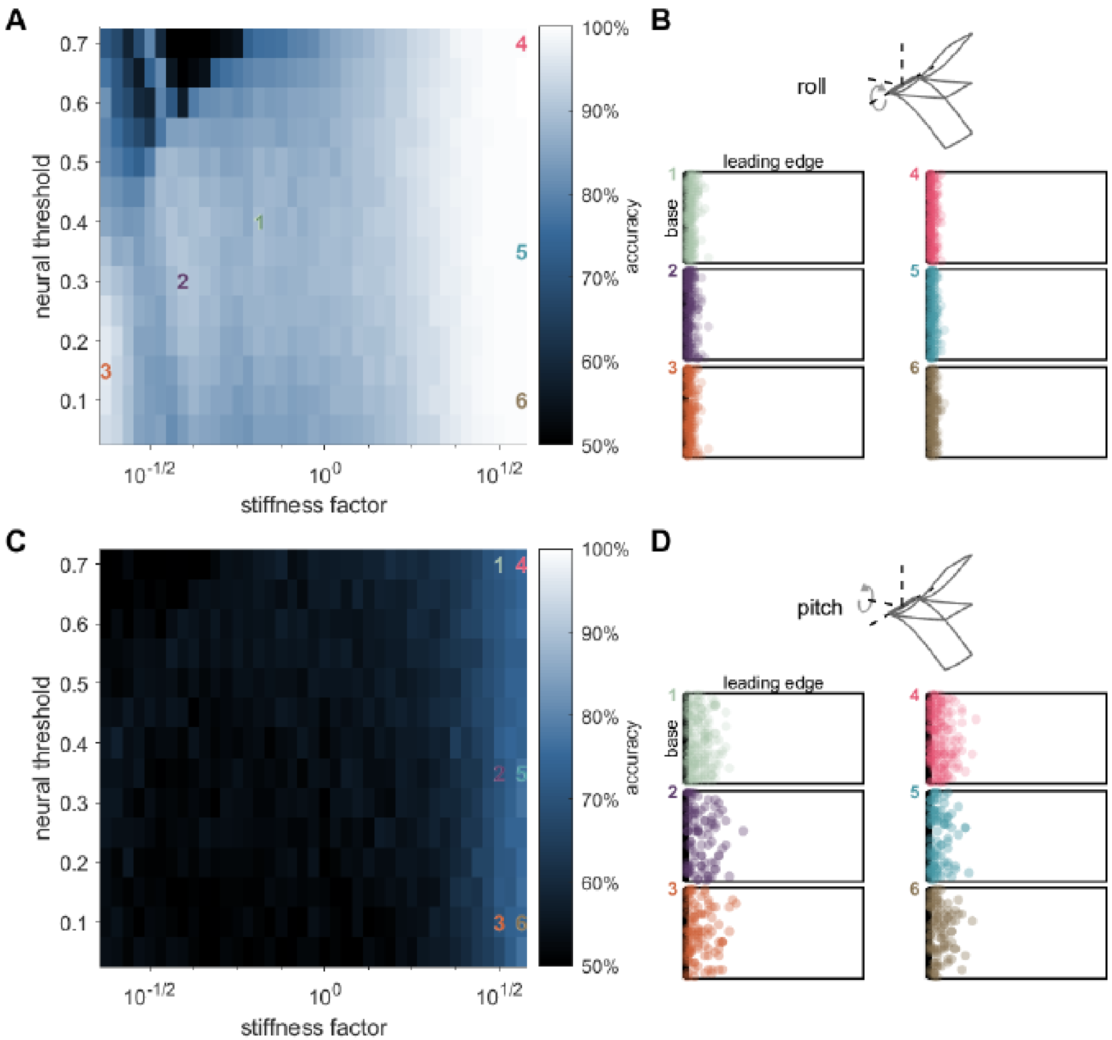

Fig 4. For detecting rotation in the roll and pitch axes, sensor locations do not depend on stiffness or neural encoding properties. A,C: Accuracy as a function of neural threshold and wing stiffness for roll and pitch, respectively. B,D: Optimal sensor locations of 10 best sensors, overlaid for 20 different data sets. Neural threshold and wing stiffness of each panel are indicated by the corresponding colored number in A. *Top:* Schematics indicate the axis of rotation.

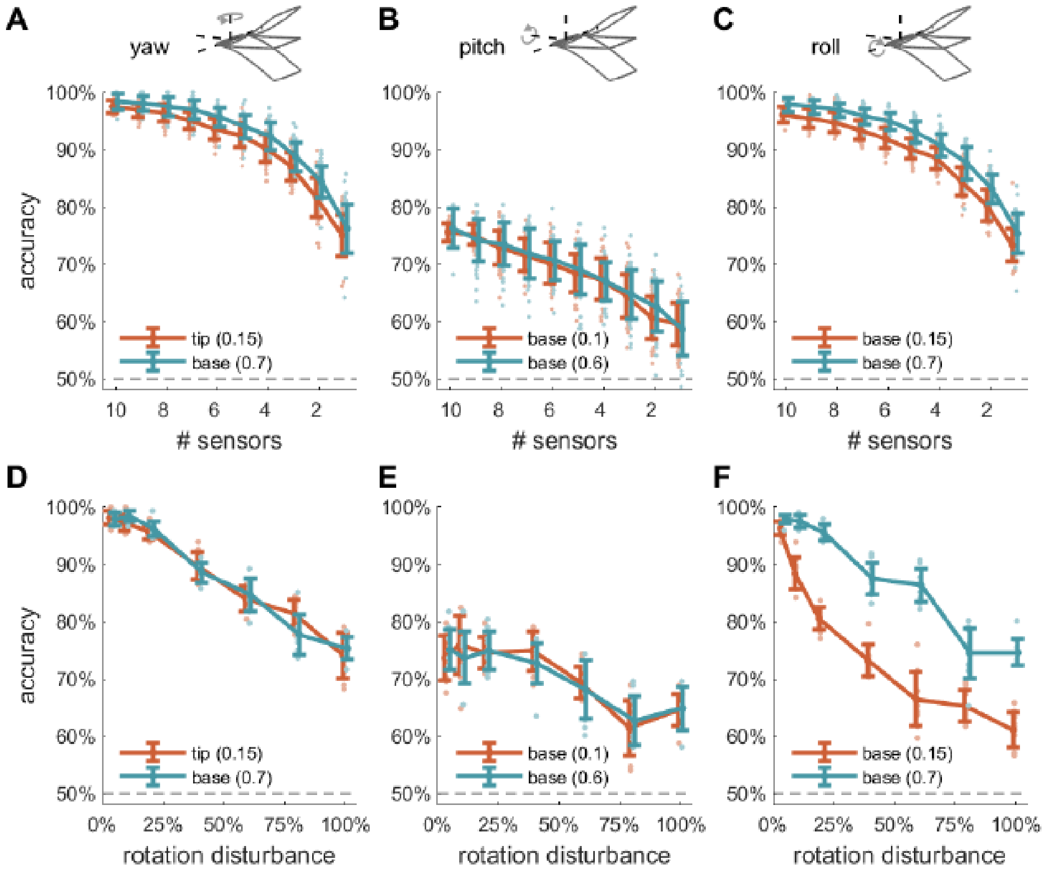

Fig 5. Robustness to sensor dropout and external disturbance. A: Accuracy of detecting rotation in the yaw axis as a function of number of sensors for two different wing stiffness/neural threshold parameter combinations (orange and teal). One parameter combination results in sensors at the wing tip, and a second results in sensors at the wing base, both with accuracy of 98% for 10 sensors. B–C: Same as A, for pitch and roll, respectively. In each case, two different parameter combinations were again chosen that produce similar accuracy with differing neural thresholds. For pitch-axis and roll-axis rotation detection, all sensors are located at the wing base. D–F: Accuracy as a function of the standard deviation of rotation disturbance for yaw, pitch, and roll, respectively, shown as the percentage of the constant rotation velocity to be detected. Parameter combinations same as corresponding A–C panels. Error bars in all panels denote ±1 standard deviation.

Line 101 – with 10 sensors… where in Fig 2 or elsewhere is the support for the number of sensors? I had to reach a later section in the paper to understand that these are the 10 best sensors. Maybe revise to “with as little as 10 sensors”

Thank you for pointing us to this potential source of confusion. We now indicate earlier in the manuscript that we generally use 10 sensors and have included additional explanation of this choice in the Methods. We also changed the indicated line as suggested.

Fig 1A, 3B, 4B sensor location. To prevent doubt please indicate the tip and base of the wings and the leading and trailing edges.

We have added these labels to all figures showing sensor placement (Figures 1-4).

The format in the references list is not uniform (see for example #10)

We believe all formatting issues in the references have been corrected.

Reviewer #2:
In this manuscript, the authors build upon previous work on the dual role of the wing as an actuator and a sensor. They do this by using computational models to investigate the effect of wing stiffness on sensing body rotations. The core aspects of the methodology were developed in previous papers (Euler-Lagrange flapping wing model-Eberle et al., 2015; optimal sensor locations-Mohren et al., 2018). Here, the authors vary the wing stiffness of the wing model while subjecting it to yaw and pitch body rotations and determine optimal sensor locations for sensing these body rotations. They then quantify the sensors' ability to classify these body rotations and test their robustness to external disturbances.

Overall, I felt the approach to be very interesting and to have a lot of potential to shed light on the role of the wing in sensing. Investigating the role of wing-stiffness and comparing optimal locations for sensing different body rotations are concrete steps towards this goal. However, I feel that the ideas that form the core of the manuscript were not adequately developed (reasons given below). My concerns are all potentially addressable and are aimed at improving the manuscript.

Thank you for this feedback and your detailed suggestions. We have now performed and included several new analyses in the revised manuscript based on your suggestions, including roll detection and sensing with both wings. We have also revised the Discussion to more directly address the implications and caveats of our work. Please see our more detailed responses below.

1. Key idea 1: wing stiffness. Because stiffness varies across flying insects, how this property effects the strains sensed by the sensors is of crucial importance. The stiffness range tested in the manuscript was centered around the stiffness of a hawkmoth's wing. However, it was not clear what determined its boundaries and how it related to the diversity of wing stiffness seen in insects. Because of this, I felt a disconnect between the simulations and its implications on "sensing strategies in insects". Bridging this disconnect would improve the manuscript by bringing out the key idea clearly (and

maybe provide testable predictions). Here are a few possible starting points to bridging this gap:
- Use a wing stiffness range that is heavily based on experimental data; something similar to the luminance scale shown in Fig. 1A in Sponberg et al., 2015 and the range occupied by hawkmoths.

Indeed, the range of stiffness that we tested was based on experimental measurements (from Combes & Daniel 2003a,b; references [28-29] in manuscript), though the range we could explore is somewhat restricted by computational limitations of the model. In particular, at low flexural stiffness values, numerical convergence became an issue. We have added text to both the Results and Methods to clarify how the range we examined relates to that in *Manduca sexta.*

Results (lines 88-91):

> The range of wing stiffness values examined centers on the average reported flexural stiffness for hawkmoth forewings (Methods) [Combes & Daniel, 2003a; Combes & Daniel, 2003b].

Methods (lines 349-357):

> The range of explored modulus values is approximately centered at 3 GPa (stiffness factor = 1), corresponding to a spanwise flexural stiffness of ~$1.5*10^{-4}$ Nm^2, comparable to average experimentally measured spanwise flexural wing stiffness in *Manduca sexta* [Combes & Daniel, 2003a]. We vary Young's modulus values from 0.7 GPa to 10.0 GPa (flexural stiffness $3.6*10^{-5}$ to $5.6*10^{-4}$ Nm^2). This approximately corresponds to the flexural stiffness range from half the wing span to the wing base [Combes & Daniel, 2003b]. Below 0.7 GPa, simulations did not achieve appropriate levels of numerical convergence, and more flexible wings therefore cannot be tested with this model.

- It might be possible to obtain high accuracy for different stiffness values by varying the neural threshold (at least for the wing stiffness factor below 1). I felt this to be an interesting aspect worth investigating further, especially in the context of using high neural threshold as way of dealing with low stiffness factor wings.

Thank you for this comment. Indeed, this interplay of stiffness and neural threshold is the key aspect of the work presented here — specifically, that neural threshold interacts with wing stiffness to determine accuracy. This interaction is particularly complex for the case of yaw detection (Figure 3). As this figure shows, with a range of neural thresholds on the vertical axis and a range of stiffness values on the horizontal axis, an intermediate neural threshold provides best performance for the lowest stiffness wings tested. We agree that this is an interesting point and have further emphasized it by adding the following to the Results (lines 117-120):

> These results suggest that classification performance for a given wing stiffness might be improved by altering the neural threshold. We next examine how neural encoding properties interact with wing stiffness by simultaneously varying both wing stiffness and the threshold of the nonlinearity in neural encoding.

- Spike timing precision in the context of stiffer wings (Line 103-111) is another interesting aspect that can be investigated, especially in the context of fast flapping insects with stiff wings. I feel that this is should be developed further.

We share the reviewer's interest in investigating spike timing precision. In our paper, the phenomenon that spike timing precision *per se* can be less important than the time between firing events is illustrated for stiffer wings in Fig 2, and this phenomenon can also occur in the case of more compliant wings. More precisely, this transition between different firing events can arise when the neural threshold closely matches the magnitude of strain fluctuations; this can lead to subtle differences in strain translating to large changes in the timing of spikes. The trajectory of the wingstroke will play a critical role in determining the timing of these distinct firing events in different sensors. We agree that this is an interesting area of future work and have now included these additional sentences in the Discussion (lines 266-272):

> In the current work, we see that the wingstroke, modeled as a sum of sines as in previous work [Eberle et al., 2015; Mohren et al., 2018], drives two distinct response events over the course of single wingbeat (Fig 2). Interestingly, these response events interact with spike timing precision in sometimes counterintuitive ways, such that lower spike timing precision can sometimes yield greater discriminability. More complex wingstroke kinematics likely play an important role in determining the phase (or phases) of the wingstroke when each neuron is most sensitive.

- I felt that there is a need to compare the model-based optimal sensor positions for different wing stiffnesses with those seen in insects. Do the simulations suggest a shift in strategies? An explicit discussion on this aspect would improve the manuscript.

Thank you for this feedback. To address this point specifically, we have added a subsection to the Discussion ("Strain sensor placement in insect wings," beginning line 240) that describes known features of campaniform sensilla placement that are common across many families of insects and compares these general trends to the results we obtain from our model:

> **Strain sensor placement in insect wings**
>
> Insect wings exhibit widely varying patterns of mechanosensor placement on their wings [47-51]. Even so, we have little understanding of the functional consequences of this diversity. Moreover, comparison between species is complicated by the vast differences in wing morphology and stiffness [29, 52]. Despite these differences, some generalizations can be made about the placement of strain sensors (campaniform sensilla). Perhaps the most striking commonality across diverse taxa is the relative abundance of campaniform sensilla near the wing base [47-49, 51, 53-55]. Taken together, the results in the present work suggest that clustering sensors at the wing base may serve as a catch-all strategy, favorable across a wide range of wing and neural encoding properties to detect rotations about multiple axes (Figs 3,4; Supp Fig 6).

<u>2. Key idea 2: sensor positions for different axis body rotations.</u> Because these sensors groups need to sense body rotations along multiple axes, it was interesting to see optimal sensor positions being computed for yaw and pitch. I was a bit surprised that roll was not added and think it should be included. In addition, I felt that there is a need to pool these sensor positions and come up with a minimum group of sensors needed to sense yaw, pitch, and roll. The supplementary had a short note on the simultaneous classification of yaw and pitch, which should be added to the main results and elaborated a bit more as it suggests that accurate classification requires different groups of sensors. Perhaps the authors could add a note on how three groups of optimal sensors, tuned for yaw, pitch, and roll, respectively, would respond to a perturbation along an intermediate axis. This is important because active body rotations might occur along these axes but external perturbation-induced rotations need not.

The reviewer makes an excellent point here, one that was also made by Reviewer 1. In response to this comment, we have undertaken a series of new analyses, and the new results are now included in the revision. We are particularly grateful for this suggestion, as we feel the revision presents a more complete and nuanced set of results that have strengthened the overall paper.

Specifically, we have made the following changes based on this feedback:

1) We have added new results for roll detection to the manuscript (Figs 4-5, copied below). See also response to Reviewer #1:

    > Thank you for bringing up this new direction — we've decided to roll with it. In this revision, we have now included a set of new analyses on roll detection and arrived at some new results.
    >
    > Based on some preliminary work in non-spiking sensors (similar to Supp Fig 3C), we had previously believed that detecting roll would be a much more challenging task than detecting pitch or yaw because the deformation modes are more similar to, and weaker than, those produced by the motion of wing flapping. Prompted by your question, as well as the suggestion of Reviewer #2, we have revisited roll detection in a more systematic way and have found that roll can indeed be detected with high accuracy. We have added these new results in the revised manuscript (Figs 4-5; Supp Figs 3-6) and believe that this strengthens the work by providing a more complete picture of rotation detection.

2) We have solved a new sensor optimization task for rotation about an axis intermediate to yaw and roll (also called "Dutch roll"). This result is included as a supplementary figure (Supp Fig 6), which is referenced in the Results (lines 168-172) and described in Supporting Information as follows:

    > To determine whether sensing strategies would be fundamentally different for rotation about an intermediate axis, we performed a separate set of optimizations to identify rotation about an axis intermediate between yaw and roll (equivalent to

simultaneous rotation about the yaw and roll axes). The magnitude of rotation about this intermediate axis was identical to elsewhere in the present work (10 rad/s). Rather than showing fundamental differences in performance or sensing strategies, results reflect a combination of the strategies for sensing yaw only and pitch only. Accuracy is high across a larger range of the stiffness factor/neural threshold space than for either yaw or pitch alone, with stiffer wings showing sensing strategies (all base sensors) and accuracy similar to roll detection and more compliant wings showing sensing strategies (either tip or base sensors) and accuracy similar to yaw detection (Supp Fig 6). For some parameter combinations, sensing strategies reflect a combination of the individual yaw and roll strategies (e.g., #2 in Supp Fig 6 shows both base and tip sensors).

3) We have re-done the (previously 3-way, now 4-way) classification for all axes tested and included this new result as a supplementary figure (Supp Fig 4). We interpret these 4-way classification results (and the new intermediate axis results) as similar to what is expected from the individual axis cases. We have added a new paragraph to the Results to explain this interpretation (lines 168-172):

> Thus far, we have considered rotation about three orthogonal axes individually. However, an animal potentially needs to detect rotation about multiple axes, or rotation about axes intermediate to primary body axes. We assessed optimal sensing strategies in each of these cases and found that they reflect a combination of the strategies used for single-axis detection, rather than fundamentally different strategies (Supp Figs 4-6).

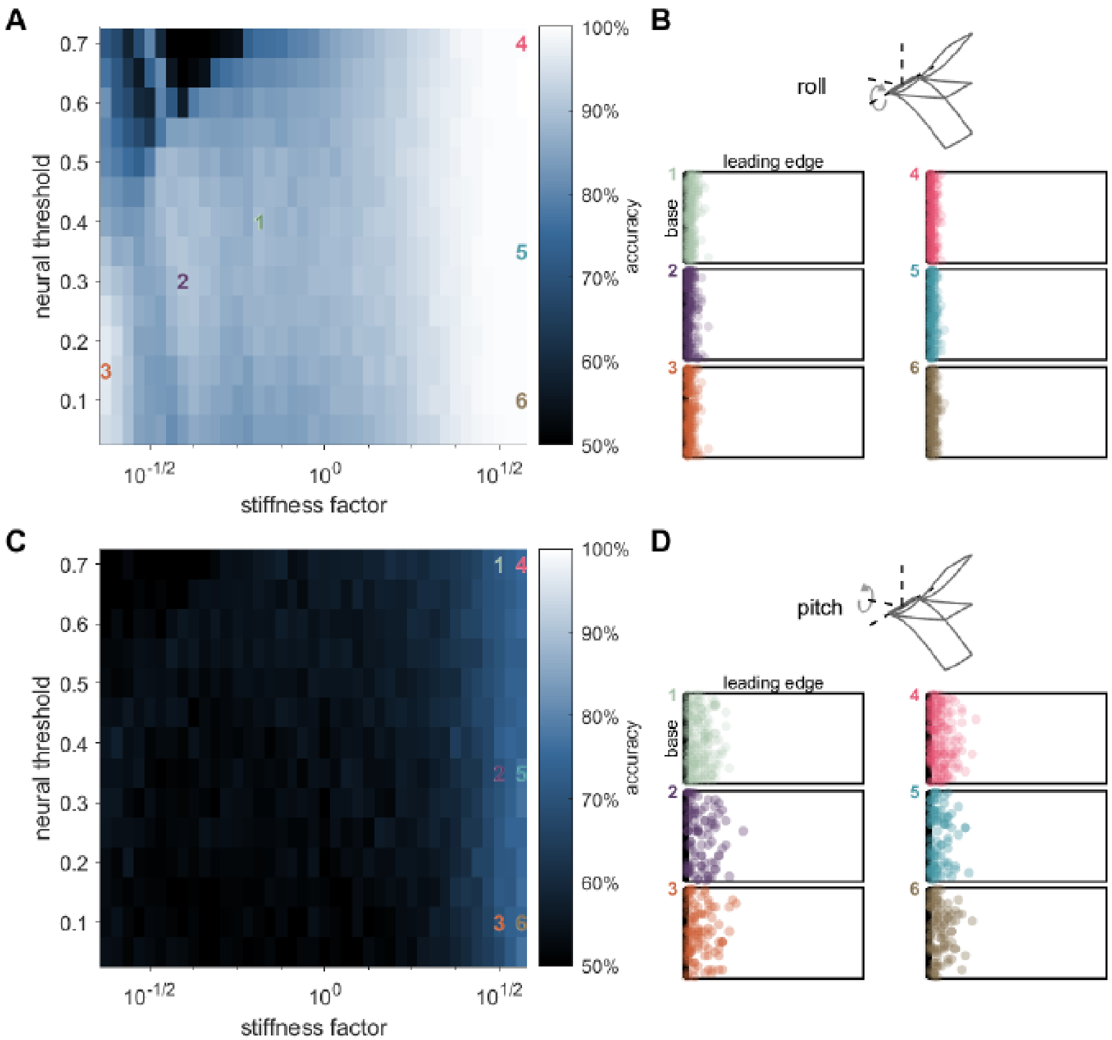

Fig 4. For detecting rotation in the roll and pitch axes, sensor locations do not depend on stiffness or neural encoding properties. A,C: Accuracy as a function of neural threshold and wing stiffness for roll and pitch, respectively. B,D: Optimal sensor locations of 10 best sensors, overlaid for 20 different data sets. Neural threshold and wing stiffness of each panel are indicated by the corresponding colored number in A. *Top:* Schematics indicate the axis of rotation.

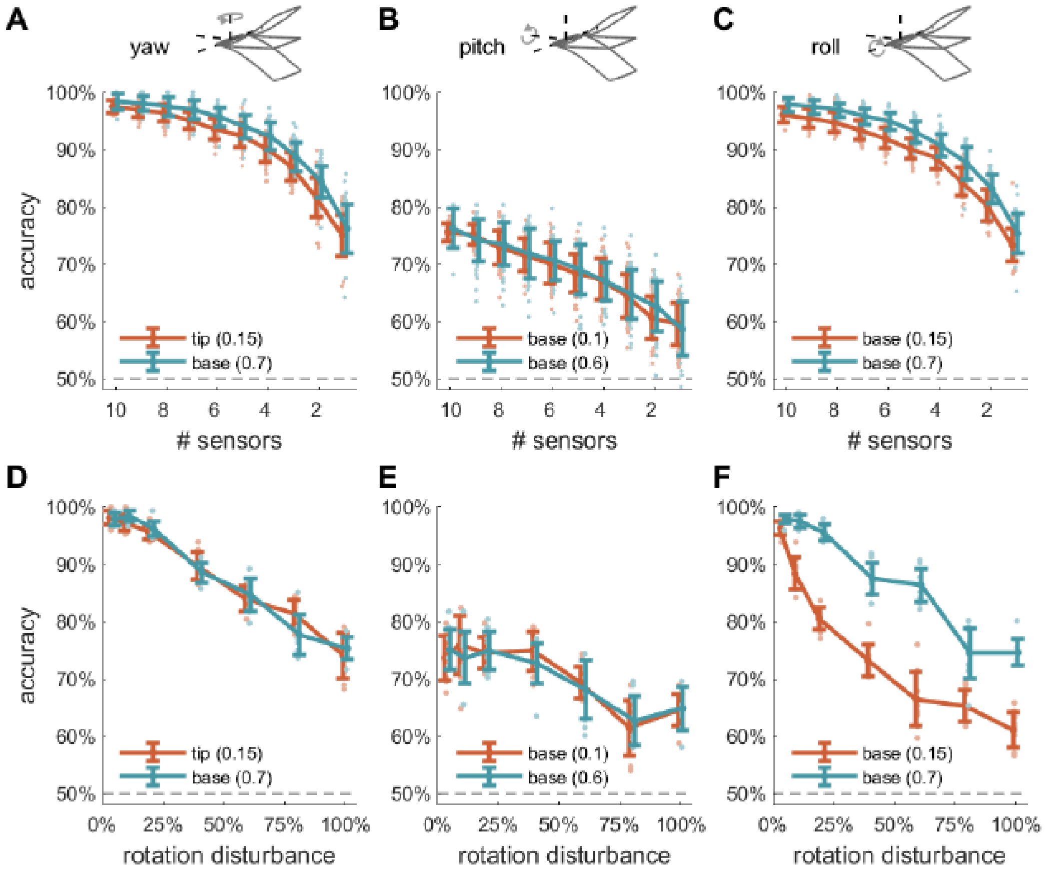

Fig 5. Robustness to sensor dropout and external disturbance. A: Accuracy of detecting rotation in the yaw axis a function of number of sensors for two different wing stiffness/neural threshold parameter combinations (orange and teal). One parameter combination results in sensors at the wing tip, and a second results in sensors at the wing base, both with accuracy of 98% for 10 sensors. B–C: Same as A, for pitch and roll, respectively. In each case, two different parameter combinations were again chosen that produce similar accuracy with differing neural thresholds. For pitch-axis and roll-axis rotation detection, all sensors are located at the wing base. D–F: Accuracy as a function of the standard deviation of rotation disturbance for yaw, pitch, and roll, respectively, shown as the percentage of the constant rotation velocity to be detected. Parameter combinations same as corresponding A–C panels. Error bars in all panels denote ±1 standard deviation.

<u>3. Caveat of the methodology:</u> the methodology used to determine sensor locations is optimized for classification, that is, to determine the presence or absence of a particular body rotation. Mohren et al., 2018 used the methodology from an engineering perspective and a bio-inspired approach. However, in this manuscript, this methodology is used to better understand wing-based sensing strategies. I feel that there is a need to talk about the caveat of this approach, especially the assumptions and limitations, in the context of insect flight.

We appreciate this comment, and the revised manuscript focuses on understanding wing mechanosensing more than our previous paper. Accordingly, we have added an additional section to the Discussion ("Additional features driving optimal sensor placement," beginning line 251) that addresses limitations of the current study and how they might be approached in future work:

Additional features driving optimal sensor placement

Insect species vary widely in features that are likely to play a role in determining effective mechanosensor placement on the wings: wing size, shape, and venation pattern, among others. Understanding the functional consequences of these different features and how they impact spatiotemporal patterns of strain on the wing will be necessary for elucidating principles guiding the differing placement of sensory structures across diverse insect taxa. Answering these questions will require different computational models that allow more flexible manipulation of these variables. For example, models based on the finite element method (FEM) support more realistic wing geometry, wing venation patterns, and non-uniform stiffness [29, 49]. The consequences of many of these properties have been previously explored in a variety of models, although none from a sensing perspective [18, 19, 29, 56, 57]. More biologically realistic features will result in more complex spatiotemporal patterns of wing strain, likely driving sensor placement to be far more complex and varied than those uncovered in the current study.

The wingstroke trajectory and kinematics may play a particularly important role in determining optimal sensor placement. In the current work, we see that the wingstroke, modeled as a sum of sines as in previous work [23, 31], drives two distinct response events over the course of single wingbeat (Fig 2). Interestingly, these response events interact with spike timing precision in sometimes counterintuitive ways, such that lower spike timing precision can sometimes yield greater discriminability. More complex wingstroke kinematics likely play an important role in determining the phase (or phases) within a wingstroke when each neuron is most sensitive. The trajectory of the wingstroke may also be used as a mechanism for active sensing, in which the animal modifies its motor output to acquire more useful sensory information [58-62]. Understanding the consequences of varying motor output for incoming sensory information will be crucial to understanding sensing strategies in flight.

Although we varied neural encoding properties (nonlinear threshold and filter frequency, Fig 3, Supp Fig 1), all sensors on a given wing have identical encoding properties. Previous work suggests that there are likely two sub-populations of strain-sensitive neurons on the wings of moths [63]. It is possible that a wing with different sensor types may need fewer sensors and adopt a qualitatively different encoding strategy. Additionally, selectivity to multiple features has been demonstrated in neurons in multiple sensory systems, including strain-sensitive neurons in the halteres of crane flies [6, 64]. Allowing individual sensors to respond to multiple stimulus features may also allow for fewer sensors and different sensor positioning. However, it has

> recently been suggested that all strain-sensitive neurons on insect wings may, in fact, respond similarly in the context of flight (i.e., for rapidly fluctuating stimuli), suggesting that sensor placement may be the primary determinant of information encoded by a given sensor [65].
>
> The current work investigates sensing strategies by finding solutions to a set of optimization tasks. However, biological systems need not operate at any true optimum, but may instead simply need to be “good enough" or robust to failure [66]. Solving for optimal sensor locations likely explains some of the features of the present study, for example, the abrupt shift between wing base and wing tip sensing strategies for yaw detection (Fig 3). If sensor locations are restricted to either the distal or the proximal half of the wing, sensor performance does not fall off sharply at these same transitions (Supp Fig 7). We investigated robustness of the optimal sensing strategies in the current study and found little difference between different sensor properties (locations or threshold), suggesting that a need for robustness would not favor one of these strategies over another. Exploring the range of sensing strategies that meet some (sub-optimal) performance criterion may be an interesting area of future work.

Line 20-24: "This … those features." I found the sentence to be too long. I had to read it a few times before I was able to grasp the meaning. I recommend breaking it down into smaller sentences.

Thank you. We have revised this sentence as follows:

> This transformation from input to response can often be accurately represented by a two-part model of neural encoding. One part consists of a stimulus feature or features to which the neuron is sensitive, and the second part is a nonlinear function that captures how selective the neuron is for those features.

Line 98-102: "This results … ~75% accuracy." This difference in spike timing should be shown clearly, with a linked figure.

We have added insets to panels D and E of this figure that show magnified versions of the relevant time windows so that the subtle differences in spike timing can now be resolved by eye.

Fig. 2: The difference in the green shades used for rotation and no rotation is hard to perceive.

We have increased the difference between the two shades of green.

Line 109-111: "Lower precision … approaching 100%." This line seems to hint towards something interesting. It needs to be elaborated upon, maybe as a discussion point.

We found this interesting as well! To elaborate, we have included this as a point in the Discussion (lines 266-272):

> In the current work, we see that the wingstroke, modeled as a sum of sines as in previous work [Eberle et al., 2015; Mohren et al., 2018], drives two distinct response events over the course of single wingbeat (Fig 2). Interestingly, these response events interact with spike timing precision in sometimes counterintuitive ways, such that lower spike timing precision can sometimes yield greater discriminability. More complex wingstroke kinematics likely play an important role in determining the phase (or phases) within a wingstroke when each neuron is most sensitive.

Fig 3, 4: The color difference between dots is not perceptible for certain combinations. Maybe add numbers as well?

Thanks for this suggestion — we have added numbers and it does improve readability.

Line 138-141: "When the … strategies emerge." An interesting point that needs to be elaborated upon, maybe in the discussion (refer to point 1).

We have added a paragraph to the Discussion (lines 290-301) that addresses why these optimal sensing strategies may shift so abruptly from wing base to wing tip with small changes in parameters:

> The current work investigates sensing strategies by finding solutions to a set of optimization tasks. However, biological systems need not operate at any true optimum, but may instead simply need to be "good enough" or robust to failure [Marder et al., 2015]. Solving for optimal sensor locations likely explains some of the features of the present study, for example, the abrupt shift between wing base and wing tip sensing strategies for yaw detection (Fig 3). If sensor locations are restricted to either the distal or the proximal half of the wing, sensor performance does not fall off sharply at these same transitions (Supp Fig 7). We investigated robustness of the optimal sensing strategies in the current study and found little difference between different sensor properties (locations or threshold), suggesting that a need for robustness would not favor one of these strategies over another. Exploring the range of sensing strategies that meet some (sub-optimal) performance criterion may be an interesting area of future work.

Line 150-152: "First, classification … stiffest wings." I wonder how this changes when one uses two wings to classify rotation instead of one. Pitch/roll related effects, at least with halteres, are ambiguous when one uses information only from one haltere. Adding two wings to the simulation is something the authors should consider. It adds a lot more work to the revision, but I feel it will give more biologically relevant insights.

Because the wings we study are symmetric in both shape and stiffness, sensing strategies for two wings are expected to be approximately the same. However, we followed the reviewer's suggestion to determine whether accuracy would be significantly impacted by using two wings to perform the yaw sensing task.

In a new analysis, we hold the total number of sensors constant at 10, though now sensors may be distributed across the two wings. If there are a small number of locations that are particularly informative, addition of the second wing could significantly boost performance. However, we see only modest gains in accuracy for a subset of stiffness/neural threshold combinations. For simplicity, we therefore focus on sensing in a single wing. We have added this explanation along with an accompanying figure (Supp Fig 2) to the Supporting Information. We have also added the following to the Results (lines 150-152):

> Sensing accuracy and sensor locations are minimally impacted if sensors are allowed to be located on two identical wings, one on each side of the body (Supp Fig 2).

Line 152-155: "Second, optimal … wing base." Needs to be elaborated in the discussion (refer to point 2).

We have now added a brief additional explanation after this sentence (lines 162-164):

> This observation is consistent with the fact that twisting modes elicited by pitch and roll are much smaller or nonexistent, respectively, compared to yaw.

The prevalence of sensors at the wing base for both roll and pitch is also addressed in the new subsection to the Discussion ("Strain sensor placement in insect wings," beginning line 240).

Discussion: In general, I found the discussion to be too broad and not adequately linked to the results. Some sections were redundant with respect to points raised in the introduction (e.g. body structure transforms sensory inputs). I feel that there is a need to flush out the discussion and link it to the broader context, which in this case is the diversity of wing stiffness and sensory positions seen in insects (refer to point 1, 2). In addition, there is a need to explicitly state the caveats of the methodology (refer to point 3).

Thank you for this feedback. In our revision, we have restructured and edited large parts of the Discussion, which we believe to be significantly improved in clarity, explicit links to results, and methodological caveats.

We have removed the first subsection ("Body structure transforms sensory inputs") due to the redundancy the reviewer points out with the Introduction. As described above, we have added two new subsections: "Strain sensor placement in insect wings" and "Additional factors driving optimal sensor placement." The first focuses on briefly summarizing what is known about placement of campaniform sensilla across diverse insect taxa and pointing out the most striking correspondence between our results and what is observed in nature, namely the high concentration of strain sensors generally observed near the wing base. The second new subsection focuses on addressing caveats of our methodology that were not previously discussed at length, focusing on aspects of wing structure that are likely relevant for determining optimal placement strategies more directly comparable to insect wings. These features require different models to investigate and are an interesting area for future work.

Reviewer #3:

The manuscript explored the combined effect of structural stiffness and neural encoding on the optimal sensing of flapping flight, which is a novel and interesting topic. The reviewer thinks the quality of the manuscript meets the standard of publishing with PLOS Computational Biology. The reviewer also has several questions as follows,

The results show that when the stiffness and the neural threshold change, the optimal sensor locations are clustered either at the tip or the base of the wing during yawing motion (Fig. 3B). The reviewer is curious about what causes this abrupt change of the optimal locations from one side of the wing to another. What might be the underlying physics that may explain this change? The reviewer is also curious about whether there is a scenario when the optimal locations of the sensor are located away from the four corners of the rectangular wing. A finer parametric study of the stiffness–neural threshold space may help answer this question.

The reviewer asks an incisive question about the physical foundations of our results, so we will address it in some detail. Although the abrupt change of sensors from wing base to wing tip in the case of yaw detection might at first seem counterintuitive, it is somewhat less surprising when considering the strain modes that are expected to be elicited by rotation in the yaw direction. Put very briefly in the manuscript (lines 140-143): "Note that sensors are expected to fall at the wing corners, as observed in previous work [Mohren et al., 2018], because rotation elicits a twisting mode in wing bending, which leads to the largest differences in strain at wing corners between rotating and non-rotating conditions [Eberle et al., 2015]." Pictorially, the wing is expected to bend as follows (Fig. 4 from Eberle et al., 2015):

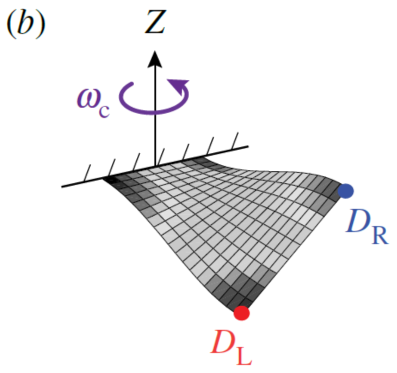

This physics explains why sensors fall in the 4 corners for the yaw detection task, though not why the optimal locations shift abruptly between the tip and base. Broadly speaking, the shift is because strain fluctuations at the wing base are significantly larger than those at the wing tip, and for different stiffness values, the neural threshold will be better matched to either the wing base or wing tip. Because our procedure is finding *optimal* sensor locations, if sensors at the wing base provide even a small advantage over sensors at the wing tip, all sensors will be placed at the wing base. This is not to say that performance will necessarily be poor with sensors in the other location. In fact, performance is often quite good even in sub-optimal

locations. To further illustrate this point, we have added a figure to the supplement (Supp Fig 7) with results for restricting sensors to either the distal half of the wing or the proximal half of the wing, which shows that performance is quite good, even with sensors that are restricted to either the base or the tips.

For these reasons, we do not expect a finer parametric study to reveal fundamentally different patterns in sensor placement. Additionally, we note that detection of rotation in the roll and pitch axes both result in optimal sensor placements near the wing base, though not restricted to the corners and showing a generally broader spread than sensor locations for yaw (Fig 4, including new results for roll axes). In other words, there are indeed cases where sensor placement does not fall at the four corners. Sensor placement is even less restricted for 4-way classification of flapping only, yaw rotation, pitch rotation, and roll rotation (new result shown in Supp Fig 4).

We anticipate that future work to incorporate additional details of biologically realistic wings will further complicate the resulting spatiotemporal patterns of strain in the wing, which will likely result in even more varied placement for optimal sensors. We have added a new subsection to the Discussion ("Additional factors driving optimal sensor placement") that describes some of the limitations of our current modeling approach and how we might expect results to change based on incorporation of more biological realism.

Can the authors try to explain why in the pitching motion, the accuracy is much reduced and the optimal sensor locations are less clustered when compared with those of the yawing motion?

For the pitching motion, far less twisting is elicited in the wing than for yaw, as depicted above in the figure from Eberle et al., 2015. We have now noted this in the paper (lines 162-164):

> ...optimal sensors are located near the wing base for roll and pitch for all parameters tested (Fig 4B,D). This observation is consistent with the fact that twisting modes elicited by pitch and roll are much smaller or nonexistent, respectively, compared to yaw.

The twisting mode is therefore not useful for detecting pitching (or rolling, as shown in the new results) and so sensors are not preferentially placed in the wing corners. The fact that strain fluctuations are largest at the wing base likely plays a role in this being an optimal location for sensors in the cases of pitch (and roll).

The rotations in this manuscript (yaw and pitch) are performed at constant angular speed. Since insects often perform maneuvers with accelerations, will the current methods be able to access the effect of angular accelerations?

The reviewer brings up an interesting point. While we acknowledge that realistic maneuvers are performed with accelerations, the model we use in this work can only generate strain patterns for constant angular rotations, so we cannot address this point directly in the scope of the current work. We may hypothesize that an animal could detect accelerations by detecting a difference in rotation from one wingbeat to the next. In that sense, the current framework could indeed be used to detect acceleration if the preceding wingbeat (or multiple wingbeats) had

been identified as “no rotation” cases and the current wingbeat is classified as a “rotation” case. However, this scenario does not take into account potentially interesting transient effects in wing deformations that could result from acceleration. This is an exciting and open area of future investigation.
